# Comparison of active learning algorithms in classifying head computed tomography reports using bidirectional encoder representations from transformers

**DOI:** 10.1007/s11548-024-03316-7

**Published:** 2025-01-08

**Authors:** Tomohiro Wataya, Azusa Miura, Takahisa Sakisuka, Masahiro Fujiwara, Hisashi Tanaka, Yu Hiraoka, Junya Sato, Miyuki Tomiyama, Daiki Nishigaki, Kosuke Kita, Yuki Suzuki, Shoji Kido, Noriyuki Tomiyama

**Affiliations:** 1 Department of Radiology, Osaka University Graduate School of Medicine, 2-2, Yamadaoka, Suita, Osaka, 565-0871 Japan; 2 Department of Artificial Intelligence Diagnostic Radiology, Osaka University Graduate School of Medicine, 2-2, Yamadaoka, Suita, Osaka, 565-0871 Japan; 3 Department of Diagnostic Imaging, Osaka General Medical Center, 3-1-56. Mandai Higashi, Sumiyoshi, Osaka, 558-8558 Japan; 4 Department of Diagnostic Radiology, Sakai City Medical Center, 1-1-1, Ebaracho, Sakai, Osaka, 593-8304 Japan; 5 Division of Health Science, Osaka University Graduate School of Medicine, 1-7, Yamadaoka, Suita, Osaka, 565-0871 Japan

**Keywords:** Natural language processing, Active learning, Bidirectional encoder representations from transformers, Radiology report, Medical safety

## Abstract

**Purpose:**

Systems equipped with natural language (NLP) processing can reduce missed radiological findings by physicians, but the annotation costs are burden in the development. This study aimed to compare the effects of active learning (AL) algorithms in NLP for estimating the significance of head computed tomography (CT) reports using bidirectional encoder representations from transformers (BERT).

**Methods:**

A total of 3728 head CT reports annotated with five categories of importance were used and UTH-BERT was adopted as the pre-trained BERT model. We assumed that 64% (2385 reports) of the data were initially in the unlabeled data pool (UDP), while the labeled data set (LD) used to train the model was empty. Twenty-five reports were repeatedly selected from the UDP and added to the LD, based on seven metrices: random sampling (RS: control), four uncertainty sampling (US) methods (least confidence (LC), margin sampling (MS), ratio of confidence (RC), and entropy sampling (ES)), and two distance-based sampling (DS) methods (cosine distance (CD) and Euclidian distance (ED)). The transition of accuracy of the model was evaluated using the test dataset.

**Results:**

The accuracy of the models with US was significantly higher than RS when reports in LD were < 1800, whereas DS methods were significantly lower than RS. Among the US methods, MS and RC were even better than the others. With the US methods, the required labeled data decreased by 15.4–40.5%, and most efficient in RC. In addition, in the US methods, data for minor categories tended to be added to LD earlier than RS and DS.

**Conclusions:**

In the classification task for the importance of head CT reports, US methods, especially RC and MS can lead to the effective fine-tuning of BERT models and reduce the imbalance of categories. AL can contribute to other studies on larger datasets by providing effective annotation.

**Supplementary Information:**

The online version contains supplementary material available at 10.1007/s11548-024-03316-7.

## Introduction

In recent years, the problem of missed radiological findings by physicians has become a social issue, which sometimes leads to significant disadvantages for patients, such as the progression of malignant diseases or sometimes mortality. Systems using natural language processing (NLP) is expected to enable the extraction of important findings and automatically alert the physician [1]. However, the development of such systems requires a large amount of train data annotated by the human doctors, which is a barrier to the research. Especially when a large number of unlabeled data are available, whereas the labeling cost is high, prioritizing the data to be labeled can lead to efficient learning of NLP models.

Active learning (AL) is a subfield of artificial intelligence based on the hypothesis that when a model is allowed to choose the data by which it is trained, it will perform better with less train data [2, 3]. Among many scenarios of AL, pool-based sampling [4] is commonly used. In general, models trained on a limited amount of labeled data perform inference on unlabeled data. Based on the model’s output, *informative scores* are computed, and by selecting those with high scores, the data that should be labeled are determined. This is applied especially when there is a small set of labeled and a large pool of unlabeled data available. Typically, instances to be annotated are extracted from the pool according to the informativeness to the model [2]. The usefulness of AL has been demonstrated in such fields as image classification [5]. Research applying AL to NLP tasks has been performed [6], before [7] and after [8, 9] the advent of the pre-trained model called Bidirectional Encoder Representations from Transformers (BERT) [10]. In the field of medical research, AL is used to detect depression symptoms from online forum texts [11] and classify pathology reports [12].

There have been numerous Al approaches, and, as far as we know, there is no consensus on which is the best method. Thus, using a database for head computed tomography (CT) reports, this study aims to explore the benefits of AL for the classification of report importance. Specifically, we investigate whether the performance of NLP improves when using a small subset of the dataset. In addition, since AL may assign high informative scores to outliers or anomalous data, contributing to inefficient training, we also examine whether postponing annotations for a certain number of samples (referred to as *postponed case*: PC) results in better performance compared to the original AL methods.

## Materials and methods

Approval for this study was obtained from the internal Ethics Review Board of Osaka University Hospital (Suita, Japan). The need for informed consent was waived because of the retrospective nature of this study.

### Dataset

In this study, the dataset used in [1] was appropriated. This is composed of 3728 reports for plain head CT conducted at Osaka University Hospital. All of these reports were written in Japanese. These were categorized (Report Importance Category: RIC) into five categories by the agreement of neuroradiologists, according to the importance of the content: Category 0: no findings (15.0%), category 1: minor findings (26.7%), category 2: routine follow-up (44.2%), category 3: careful follow-up (7.7%), and category 4: examination or therapy (6.4%). Two thousand three hundred eighty-five reports (64%) were used as the train dataset, 597 reports (16%) were used as the validation dataset, whereas 746 reports (20%) were used as the test dataset. Since the division into these datasets was performed randomly, the proportions of each category in these datasets are imbalanced, as is the original whole dataset.

### Scenario of active learning

**Fig. 1:**
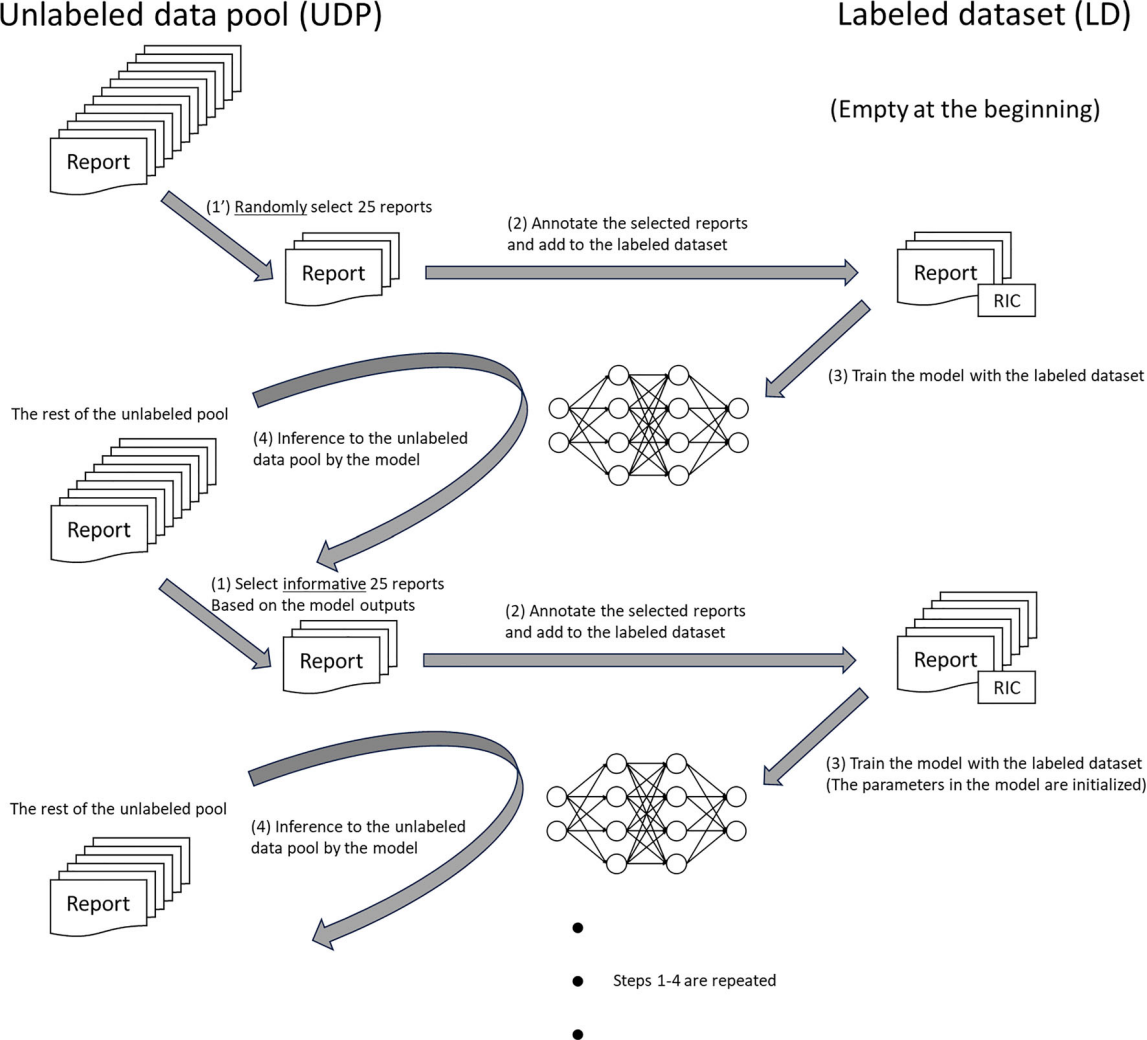
Workflow of active learning

The flowchart of the AL scenario is shown in Fig. 1. Initially, all reports in the train dataset were put into the “unlabeled data pool (UDP)”, whereas the “labeled dataset (LD)” was empty, to assume the unannotated state for all data. (1’) A fixed number (granularity, N: fixed to 25 in this research) of reports were randomly selected from the UDP and (2) added to the LD with the RIC annotation. (3) An NLP model was trained using the LD at that point. Then, (4) the rest data in the UDP were input to the trained model, creating the temporary inference. After that, (1) based on the inference, the informative score ($\varphi$) was calculated for each report using AL algorithms (described later), and the most informative N reports were selected from the unlabeled pool. Thus, steps (1)–(4) were repeated until the UDP became empty.

In this study, UTH-BERT [13] was adopted as the NLP model. This BERT model was pre-trained with medical reports written in Japanese at Tokyo University Hospital and could be regarded as a domain-specific BERT model for Japanese medical texts. A fully connected layer was used to convert the 768-dimensional UTH-BERT output (corresponding to the CLS token) into probabilities for each of the five categories. The learning rate and batch size were fixed to 2e-5 and 4, respectively, according to those used in another study [1]. The model was trained through 10 epochs and the model weights with the least loss for the validation dataset was saved.

### Comparison of active learning algorithms

Seven algorithms were used to compare the effectiveness of AL. These algorithms output IS for each report based on the inference by the model.

- Random sampling (RS)
  This is a control method. In this method, $\varphi$ are randomly selected.
  Uncertainty sampling (US) methods.
  The following four methods quantify the model’s lack of confidence in each report. In the following formulae, x represents each report used as input, and ${P(y}_{i}|x)$ the i-th highest output of the model (probability of each category) for x.
- Least confidence (LC)
  Reports with low probability for the highest category are selected.
  $$ \varphi \left( x \right) = 1 - P(y_{1} |x) $$
- Marginal sampling (MS)
  This method selects reports based on the difference between probabilities for the first and second categories.
  $$ \varphi \left( x \right) = P(y_{2} |x) - P(y_{1} |x) $$
- Ratio of confidence (RC)
  This method selects reports based on the ratio of probabilities for the first and second categories.
  $$ \varphi \left( x \right) = P(y_{2} |x)/P(y_{1} |x) $$
- Entropy sampling (ES)
  This method calculates entropy based on probabilities for each category. Originally, the higher this value is, the higher the confidence in the model’s classification can be considered; however, the minus sign has been removed from the original definition for the entropy in the following formula to give preference to those with lower values for selection.
  $$ \varphi \left( x \right) = \Sigma_{i}^{5} P(y_{i} |x) \times \log P(y_{i} |x) $$
  Distance-based sampling (DS)
  These methods use not only the data in the UDP but also those in the LD. For each report, UTH-BERT outputs a 768-dimensional vector and those vectors for the labeled data are used to calculate the category center vector (${v}_{c}$) by averaging vectors for each category. For one report ($x$) in the UDP, the distance between the corresponding 768-dimensional vector (${v}_{x}$) and each of the category center vectors is calculated and the report that gives the vector furthest from all category center vectors is selected.
  Two different methods of measuring distance were employed in this study.
- Euclidean distance (ED)
  This method calculates the distance between ${v}_{x}$ and ${v}_{c}$ using the Euclidean distance.
  $$ \varphi \left( x \right) = {\text{min}}_{c} \left\| {v_{x}  - v_{c} } \right\| $$
- Cosine distance (CD)
  This method calculates the distance between ${v}_{x}$ and ${v}_{c}$ using the cosine similarity.
  $$ \varphi \left( x \right) = 1 - {\text{min}}_{c} \left( {v_{x}  \cdot v_{c} /\left| {\left| {v_{x} } \right|} \right| \cdot \left| {\left| {v_{c} } \right|} \right|} \right) $$

For each AL method, the trial explained in the “Scenario of active learning” section was repeated ten times changing the initial LD.

### Examination of the effects of postponed case introduction

Especially in phases of AL, where the LD is small, the model may not be well trained, and truly informative data may not always be selected by the AL algorithms. In this experiment, we introduced a parameter called *postponed case* (PC), and, using the AL methods, the highest priority of PC reports was forcibly lowered, preventing those reports from being selected from the UDP.

In this experiment, due to the undesirable results of the DS methods (described in the Results section), only the four US methods were adopted, and PC was changed to 0 (original US methods), 25, 50, 75, 100, 125, and 150. The transition of performance of the models is examined.

### Statistical analysis

#### Comparison of efficacy of labeled data reduction.

To examine the AL efficacy of labeled data reduction, the minimum number of cases required to achieve certain thresholds (accuracy = 0.80, 0.81, 0.82, and 0.83) were compared. (Since seven AL methods were compared, and 10 trials were repeated for each AL method, training with all 2385 reports was conducted 70 times. As described later, since the minimum accuracy was 0.839 among the 70 trials, the maximum threshold was set to 0.83.)

#### Comparison of accuracy of NLP models with AL methods

In this research, since 2385 reports existed in the UDP initially, a model corresponding to each of the 96 steps (in the last 96th step, the remaining 10, instead of 25, cases were added to the LD) appeared. For each step, the performance of the model was evaluated using the accuracy for the test dataset and the transition of the accuracy was regarded as the result of the AL method. As described above, we performed the same trial ten times changing the combination of the initial 25 cases in the LD. For each step, the accuracies between two methods were compared using the Mann–Whitney U test, where *p* value < 0.05 was regarded as significant.

When comparing two AL methods, since there are as many as 96 steps, some of them may have significant differences while others may not, making it difficult to tabulate the overall trend, this study introduces quartiles. The learning process is divided into four groups of 24 steps, and whether each step was significant or not is examined. When comparing two methods A and B in one of the quartiles, the superiority of A over B is assessed by the difference between the number of steps in which A performed significantly better and the number of steps in which B performed significantly better. In this research, we call this value “Superiority Score (SS)”. When focusing on a single step, the probability of the occurrence of a significant difference by chance is at most 0.05, thus by solving the equation below, it can be said that A is significantly superior to B in that quartile when SS is greater than or equal to + 4. On the other hand, when SS is lower than − 4, A is regarded as significantly inferior to B.

$$ \mathop \sum \limits_{SS = x}^{24} {}_{24}C_{x} \times 0.05^{x} \times 0.95^{24 - x} < 0.05 $$

The effect of PC introduction was evaluated similarly. AL methods with nonzero PCs introduced were compared to the original (PC = 0) method.

## Results

### Comparison of transition of the accuracy among the AL algorithms

**Fig. 2:**
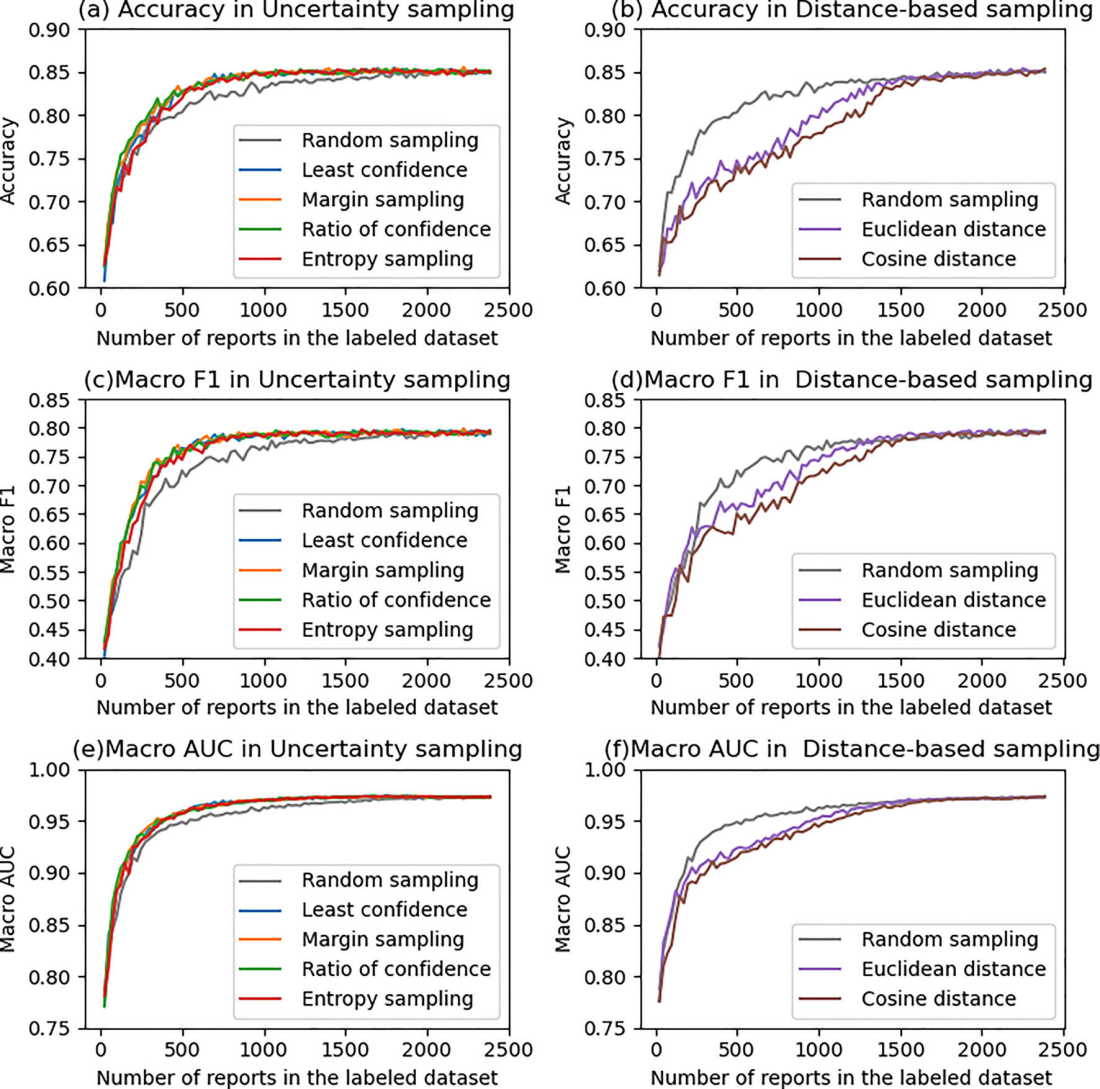
Comparison of **a**, **b** accuracy, **c**, **d** macro F1 score, and **e**, **f** macro AUC with active learning. (a, c, e) Uncertain sampling methods vs random sampling. **b**, **d**, **f** Distance-based sampling vs random sampling

**Fig. 3:**
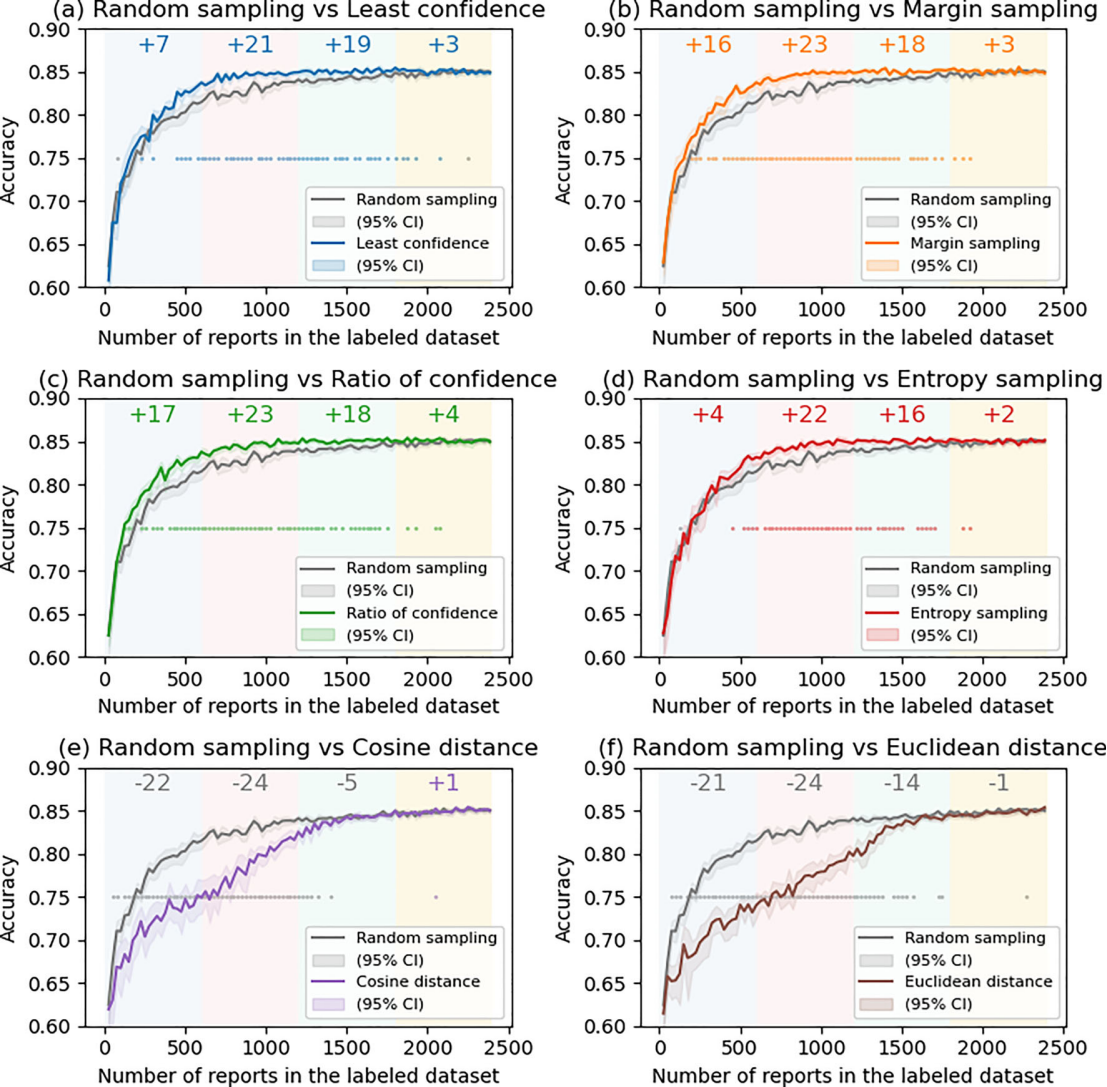
Comparison of active learning methods with random sampling. In each figure, the transition of the accuracy is shown, along with the 95% confidence interval (95% CI). Dots in the figure show significant difference (*p* < 0.05) in the step, and the color of the dots corresponds to the winner line color. The background colors show the area of quartile, and the top number shows the superiority score in that quartile. The color of the number show the line color of the winner

**Fig. 4:**
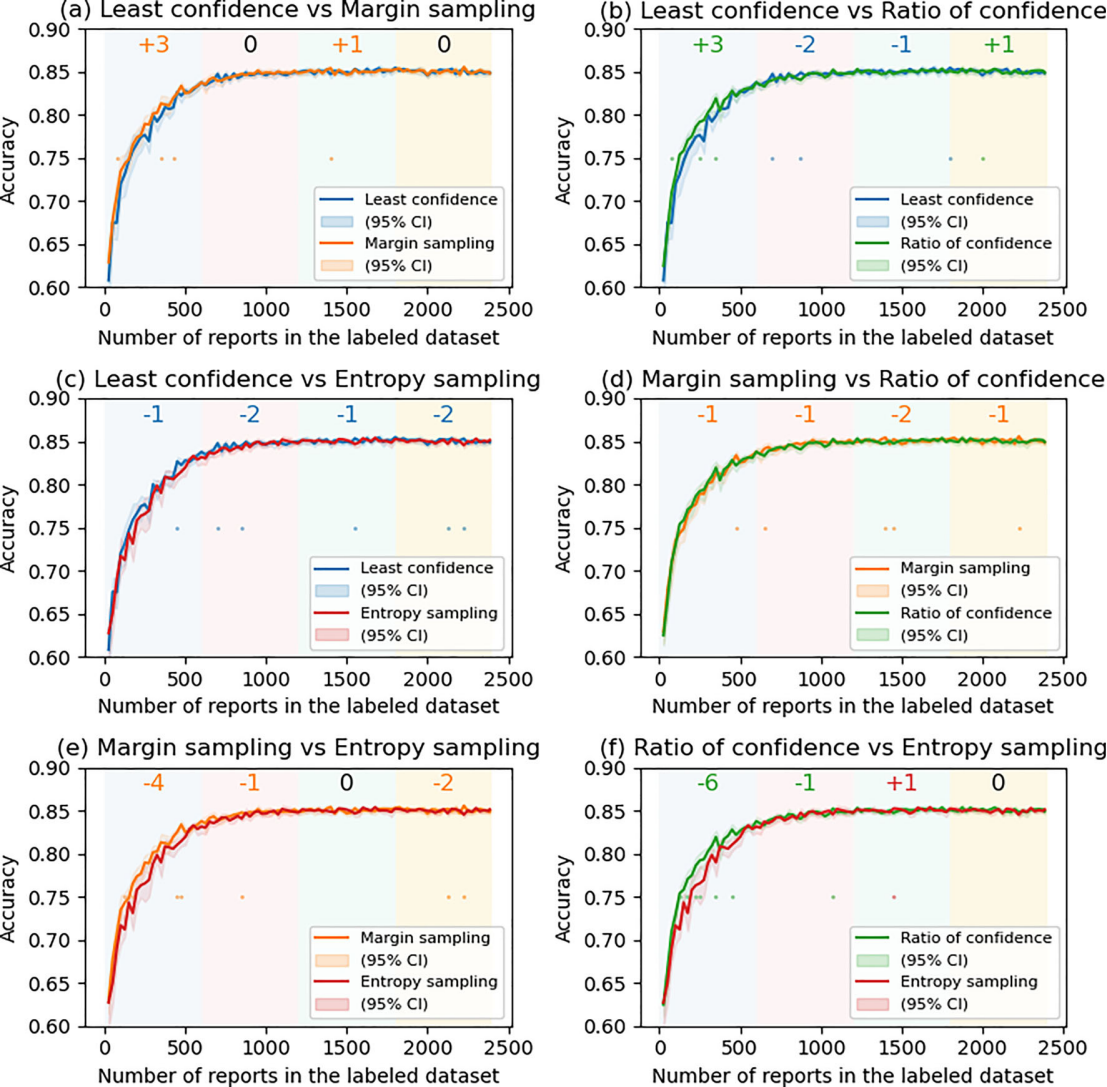
Comparison of accuracy between uncertainty sampling methods

Figure 2 illustrates the transition of the accuracy, macro F1 score, and macro (one versus rest) area under the receiver operating characteristic curve (AUC) over the test dataset. US methods tended to perform better than RS, especially when the amount of data in the LD was approximately 500–1500 by 0.01–0.03 of accuracy (Fig. 2a). In contrast, DS methods were worse than RS (Fig. 2b). Similar tendency was observed in the F1 score (Fig. 2c and d), and AUC (Figs. 2e and f). The transition of precision, recall and F1 score for each category is shown in Supplemental Material 1. In the US methods, especially in category 4, the recall tended to be higher than RS (Fig. S2i), whereas the precision was almost the same (or slightly increased) (Fig. S1i) when the LD size was 500–1500. Figure 3 shows the comparison of each of the AL methods (non-gray lines) with RS (gray line). In the figure, steps in which RS was significantly better are indicated by gray dots, and those in which AL was significantly better are indicated by dots of other colors. In addition, at the top of the figure, SS in each quartile is also described. In all US methods, SS was higher or equal to + 4 from the first to third quartile, indicating that the US methods significantly perform better than RS, whereas SS in the DS methods was lower or equal to -4, showing the significant inferiority to RS. Figure 4 shows the comparison among the US methods. In the first quartile, the accuracy of the ES was significantly lower than the MS (SS = − 4) and RC (SS = − 6), and although not significant, the ES and MS tended to perform better than the LC (SS = + 3 and + 3, respectively).

### Efficacy of labeled data reduction

The accuracy of models trained in all 2385 reports was 0.850 ± 0.005 (70 trials). Among these, the maximum and minimum accuracies were 0.863 and 0.839, respectively.

Table 1 shows the comparison of labeled data required to achieve accuracy of 0.80, 0.81, 0.82, and 0.83. In US methods, compared with RS, the amount of the requirement decreased to 59.5–84.6% (15.4–40.5% of the data were reduced), especially when the cut-off accuracy was 0.82 and 0.83, the decrease was significant. On the other hand, in DS methods, the requirement significantly increased. It is of note that in RC, although not always significant, the required data was the lowest among the models (59.5–75.0% compared with RS), which means that RC has the strongest potential to reduce the labeled data by 25.0–30.5%.

**Table 1:** Comparison of labeled data required to achieve accuracy

Accuracy		0.80	0.81	0.82	0.83
Amount of data required	RS	335.0(100.0%) ± 77.6	390.0(100.0%) ± 84.6	515.0(100.0%) ± 91.7	660.0(100.0%) ± 132.4
	LC	282.5(84.3%) ± 51.3	330.0(84.6%) ± 41.5	395.0(76.7%) ± 41.5	465.0(70.5%) ± 42.1
	MS	252.5(75.4%) ± 39.4	317.5(81.4%) ± 61.3	370.0(71.8%) ± 49.7	415.0(62.9%) ± 67.3
	RC	237.5(70.9%) ± 45.1	292.5(75.0%) ± 54.8	325.0(63.1%) ± 55.9	392.5(59.5%) ± 80.7
	ES	285.0(85.1%) ± 52.7	332.5(85.3%) ± 57.1	385.0(74.8%) ± 52.7	475.0(72.0%) ± 65.2
	ED	810.0(241.8%) ± 152.6	900.0(230.8%) ± 168.8	982.5(190.8%) ± 129.4	1170.0(177.3%) ± 82.0
	CD	1010.0(301.5%) ± 158.2	1112.5(285.3%) ± 185.8	1182.5(229.6%) ± 156.1	1317.5(199.6%) ± 98.1
*p* values	RS vs LC	0.23	0.15	< 0.01*	< 0.01*
	RS vs MS	0.02*	0.07	< 0.01*	< 0.01*
	RS vs RC	< 0.01*	0.03*	< 0.01*	< 0.01*
	RS vs ES	0.27	0.18	< 0.01*	< 0.01*
	RS vs CD	< 0.01*	< 0.01*	< 0.01*	< 0.01*
	RS vs ED	< 0.01*	< 0.01*	< 0.01*	< 0.01*
	LC vs MS	0.09	0.59	0.29	0.03*
	LC vs RC	0.03*	0.17	< 0.01*	0.04*
	LC vs ES	0.85	0.88	0.41	0.32
	LC vs CD	< 0.01*	< 0.01*	< 0.01*	< 0.01*
	LC vs ED	< 0.01*	< 0.01*	< 0.01*	< 0.01*
	MS vs RC	0.49	0.42	0.12	0.38
	MS vs ES	0.14	0.57	0.59	0.05*
	MS vs CD	< 0.01*	< 0.01*	< 0.01*	< 0.01*
	MS vs ED	< 0.01*	< 0.01*	< 0.01*	< 0.01*
	RC vs ES	0.06	0.16	0.05*	0.05
	RC vs CD	< 0.01*	< 0.01*	< 0.01*	< 0.01*
	RC vs ED	< 0.01*	< 0.01*	< 0.01*	< 0.01*
	ES vs CD	< 0.01*	< 0.01*	< 0.01*	< 0.01*
	ES vs ED	< 0.01*	< 0.01*	< 0.01*	< 0.01*
	CD vs ED	0.03*	0.04*	< 0.01*	< 0.01*

Percentages shown in the table are the comparison with RS. P-values were calculated using Mann–Whitney U test

*p < 0.05

### The ratio of RIC added to the labeled dataset

**Fig. 5:**
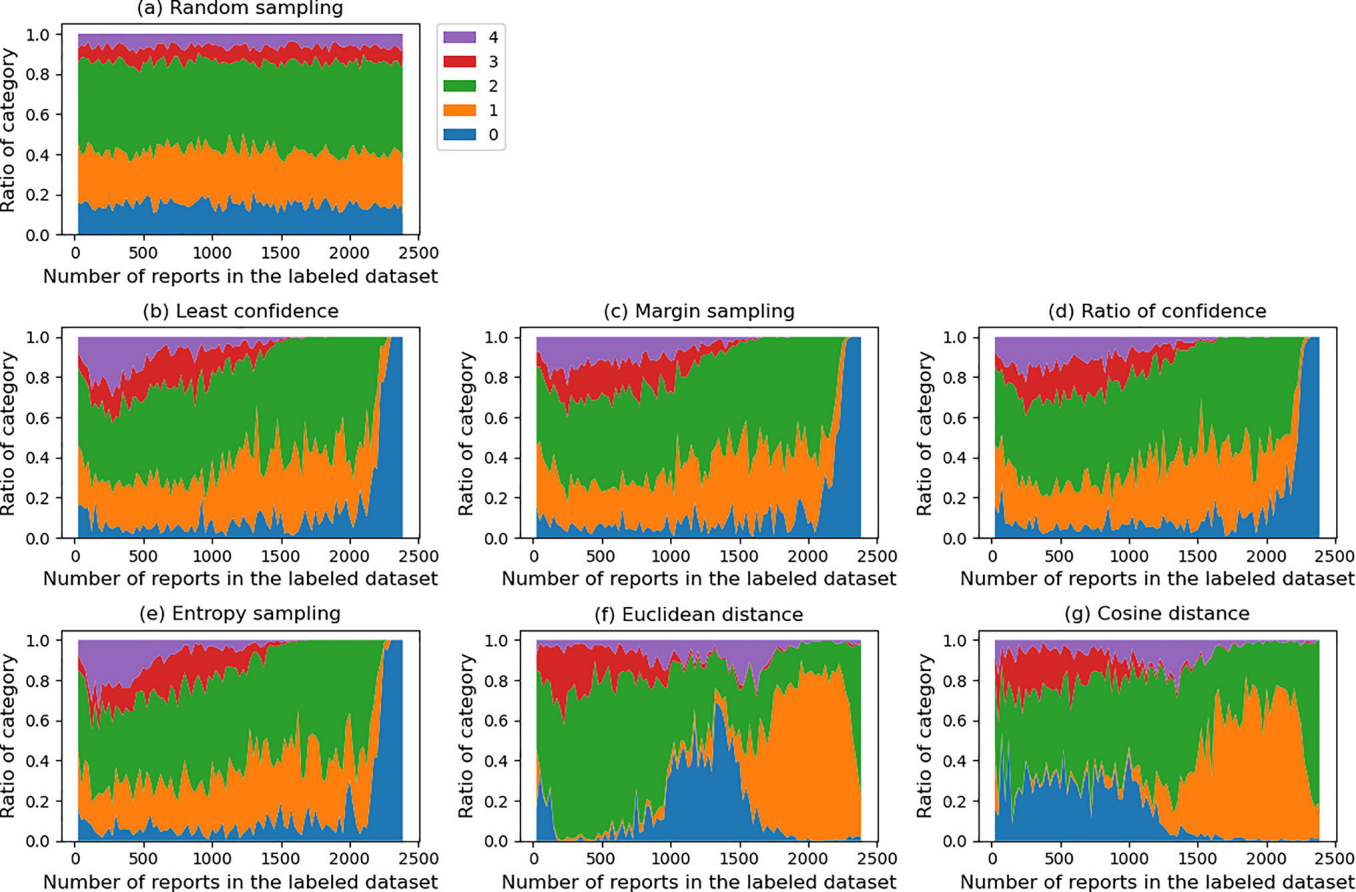
The transition of ratio of categories added to the labeled dataset in each active learning methods

Figure 5 shows the ratio of data added from the UDP to the LD in each step, by category. Focusing on the data in categories 3 and 4, which are the minority categories, they tended to be included in the LD earlier in the US methods than in the DS methods. In the US methods, almost all of these reports were added to the LD by the time when the LD size was 1500. In addition, in the US methods, category 0 data tended to remain and especially in the final step, all data added were category 0 (the category with the shortest reports), whereas in the DS methods, such a trend was not observed.

### The effects of postponed case introduction

**Fig. 6:**
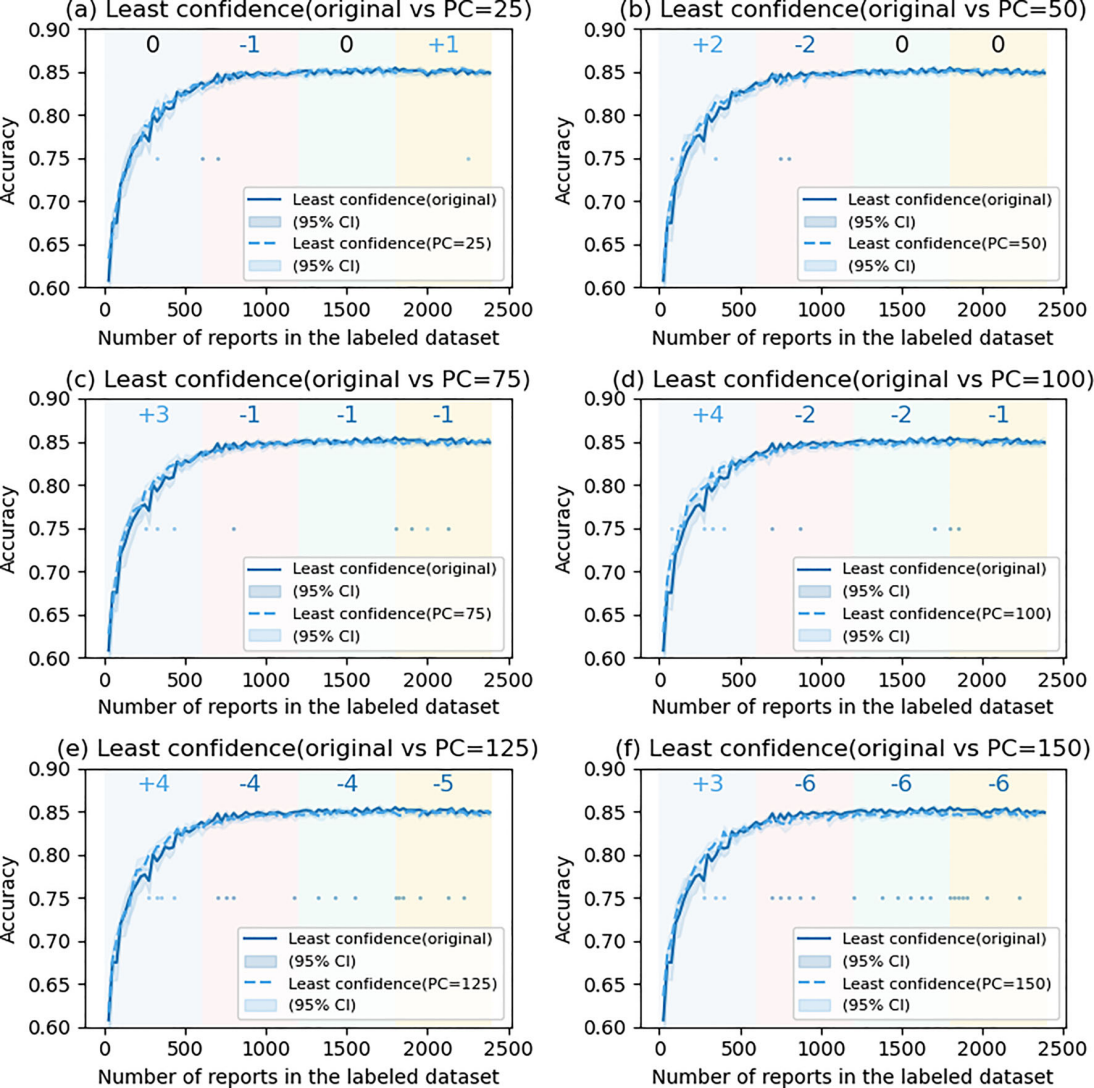
Comparison of least confidence with and without postponed case

**Fig. 7:**
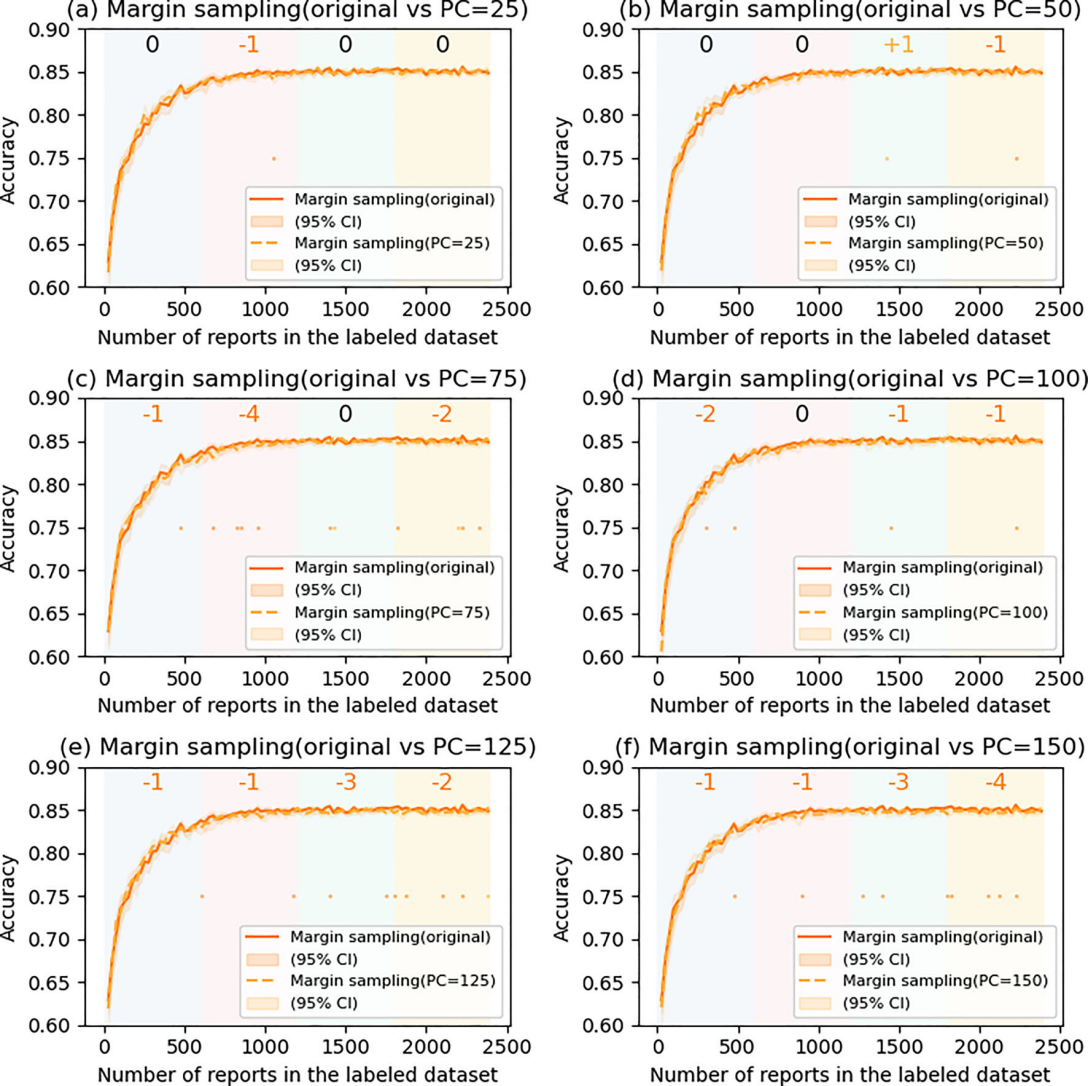
Comparison of margin sampling with and without postponed case

**Fig. 8:**
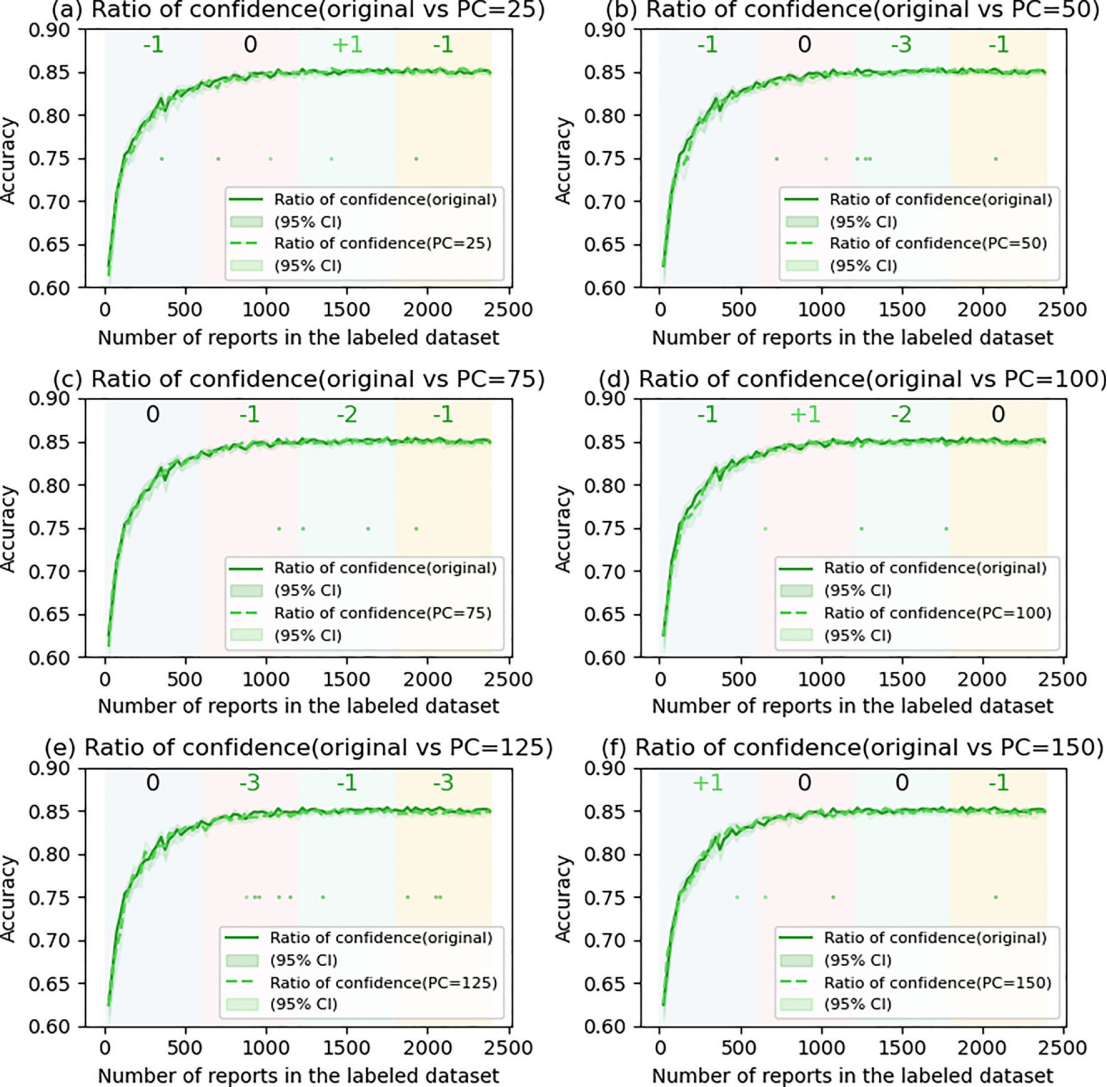
Comparison of ratio of confidence with and without postponed case

**Fig. 9:**
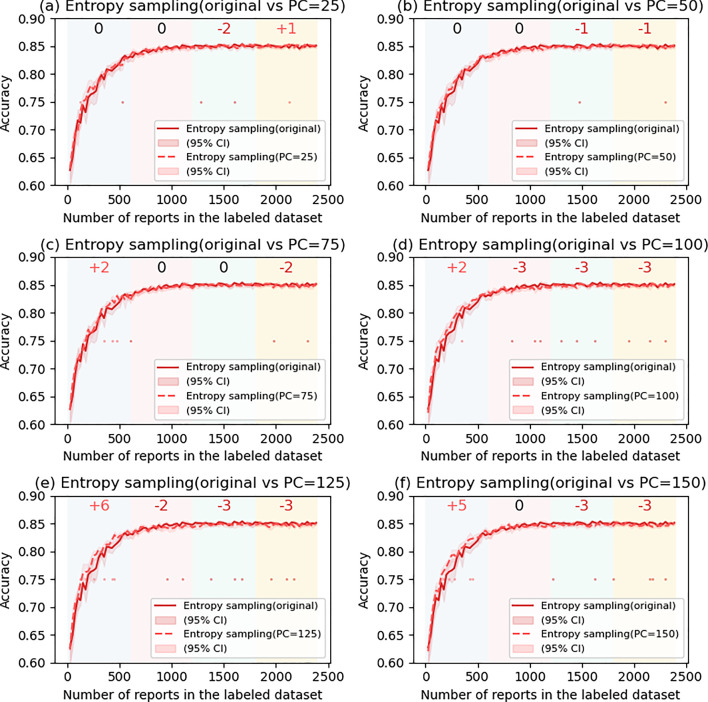
Comparison of entropy sampling with and without postponed case

Figures 6, 7, 8 and 9 show the comparison of accuracy with and without PC in US methods. In LC (Fig. 6), the performance of the models significantly improved in the first quartile when PC was 100 (SS = + 4) and 125 (SS = + 4). However, the accuracy tended to decrease in the second to fourth quartile and was significant when PC was 125. In ES (Fig. 9), the accuracy significantly increased in the first quartile when PC was 125 (SS = + 6) and 150 (SS = + 5), and tended to decrease in the second to fourth quartile, showing a similar tendency as LC. On the other hand, in MS (Fig. 7) and RC (Fig. 8), a significant increase in the accuracy with PC was not observed.

## Discussion

With the development of deep learning-based research, how to ensure the accuracy of train data is an issue. Especially in the medical field, where mistakes by AI can cause life-threatening events, data annotation should be performed by medical professionals, which is accompanied by significant costs. One of the solutions to this problem is AL. However, there have been numerous AL algorithms, and which to select is an issue. In this research, we compared six AL algorithms in terms of data reduction efficiency and transition of accuracy in the task of head CT report classification using UTH-BERT-based models.

We revealed US methods performed better than RS and DS (Fig. 2), and even among the US methods, MS and RC tended to perform better, especially when the LD size was < 600 (in the first quartile) (Fig. 3). The score in the first quartile seems important because, as shown in Table 1, the increase in the model’s accuracy was mostly observed in this area. In addition, in terms of data reduction efficiency, RC tended to perform best (not significantly different from MS) (Table 1).

In addition, reports with RIC 3 and 4 (minor categories) tended to be consumed in the earlier steps in US methods, which means that US methods have the potential to reduce the imbalance of categories. The fact that the performance for RIC 4 improved in the early steps illustrates the effects of the reduced imbalance. This study was conducted in a scenario where the dataset size was limited, and annotations by radiologists were available for all data in advance. However, in cases where annotating all data, such as all the reports from all radiology exams conducted in a particular country, is impractical, it becomes crucial to consider the efficient prioritization of data for annotation. The results of this study specifically focus on determining which AL strategy is preferable in such situations.

The effects of AL can vary according to the task, size of the dataset, NLP model, and hyperparameters of the AL (e.g. granularity). Kevin De Angeli, et al. compared the performance of AL algorithms for classifying pathology reports using convolutional neural network and showed that MS and RC performed better than the other methods when the dataset was small (1-10 K reports), whereas no “clear winner” when the dataset was large (15-162 K reports) [12]. Compared with their research, our dataset (25–2385 reports) was “very” small, and even in such situations, MS and RC outperformed the others, showing similar results. In classification tasks, where the correct answer is deterministically unique, it is crucial for NLP models not only to elevate the probability of the most likely category but also to suppress the probabilities of the other categories, specifically those ranked second, to ensure a confident decision. From this perspective, AL strategies that focus on the difference between the first and second ranked categories (MS and RC) become crucial. The results of this study support the significance of such strategies.

**Fig. 10:**
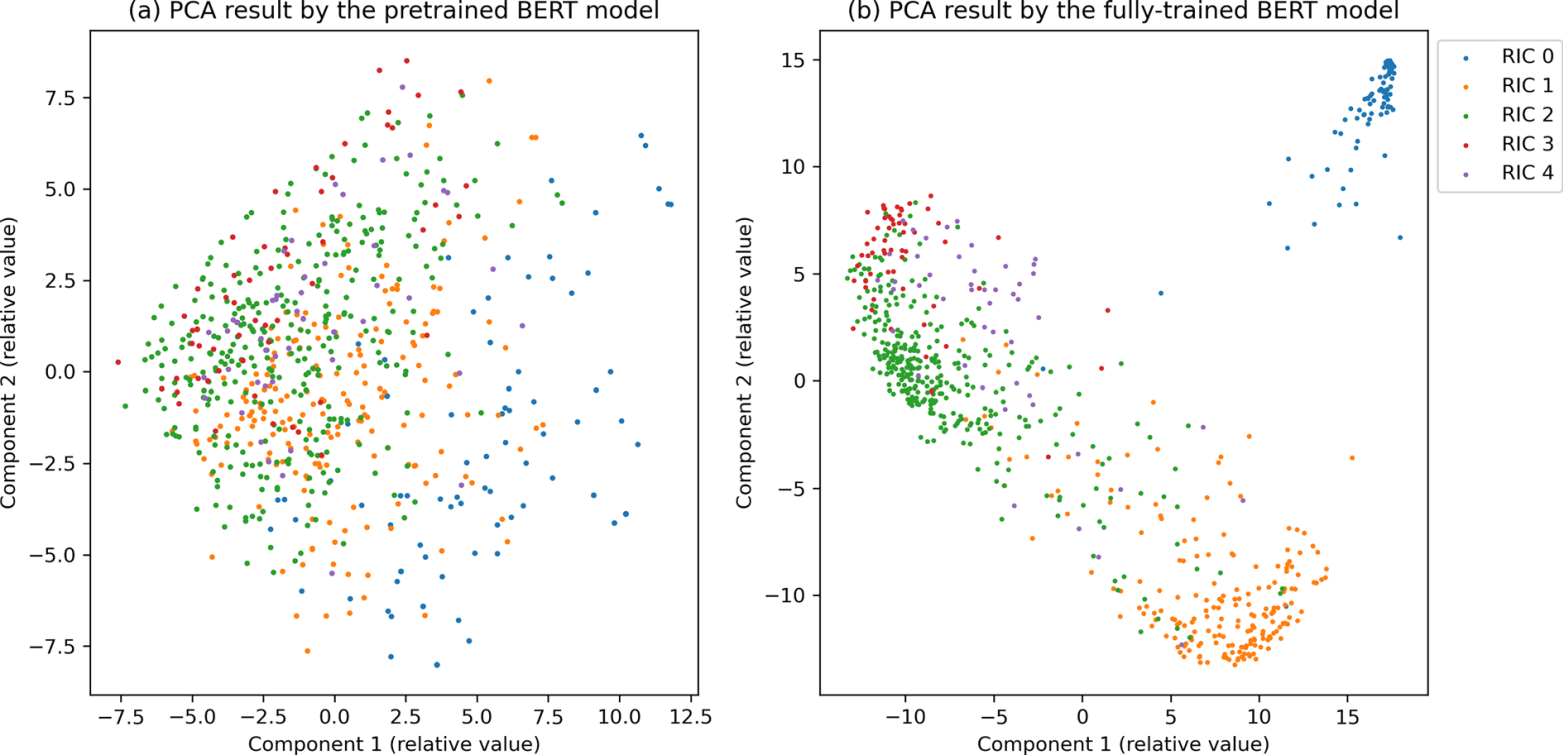
PCA results of **a** pre-trained BERT model, and **b** fully-trained BERT model for the test dataset

On the other hand, DS was inferior to US and RS methods. DS methods operate under the implicit assumption that the feature vectors by BERT should gather around their respective category center vectors (${v}_{c}$). However, we think that this assumption was not true in our analysis especially when the dataset was small. Figure 10 depicts the results of the principal component analysis, reducing the 768-dimensional vectors of BERT’s output for the test dataset to 2 dimensions. Compared with the fully trained model (Fig. 10b), the outputs by the pre-trained model (before the fine-tuning, Fig. 10a) show that the plots were scattered, and overlap is observed in the areas where the points are plotted for each category. In addition, plots for RICs 0 and 1 are sparser than RICs 2–4, which means that they are likely to be far away from vector centers, giving higher informative scores for them. Our results are consistent with the study by Kevin De Angeli, et al. [12].

Based on these results, we rank the efficiency of AL as follows:

$$ {\text{RC}} \ge {\text{MS}} > {\text{LC}} = {\text{ES}} > {\text{RS}} \gg {\text{ED}} > {\text{CD}} $$

In this study, we investigated the effects of PC introduction. To the best of our knowledge, there has been no investigation in NLP that has undertaken such considerations. Although we aimed to prevent adding outliers to the LD, the effects were limited. Our results show that induction of adequate PC (approximately 100–125) led to a significant improvement in performance for LC and ES approaches when the LD size was < 600, which implies that some of the outliers were prevented from being added to the LD. On the other hand, in RC and MS, such a phenomenon was not observed, and we think this because RC and MS themselves may be able to remove outliers. When the LD size was > 600, the performance of the model declined even with LC and ES, presumably because the introduction of PC resulted in a lack of additional informative data. Since the UDP size gradually diminishes, fixed PC can be relatively larger and larger compared with the UDP size. Based on these results, we think that although the induction of adequate PC can contribute to the increase of accuracy in LC and ES, which may contribute to the efficient training of the model, the effects depend on the PC and the final train dataset size. Thus, we believe that considering alternative methods such as MS or RC may be more efficient than introducing a new hyperparameter for AL.

There are several limitations to this study. First, the dataset used in this study was derived from one institution and the results cannot be generalizable. Second, the hyperparameters for AL, such as granularity and even the number of data in the initial UDP, were set arbitrarily, there may be more efficient values. Third, since AL methods not considered in this study, such as Query-by-committee [14], which involves training multiple NLP models simultaneously and examining the diversity of their outputs, have also been proposed, considering these approaches in future investigations may lead to even better results.

In conclusion, we revealed that in the classification task for the importance of head CT reports, US methods, especially RC and MS could lead to the effective fine-tuning of BERT models and reduce the imbalance of categories. Although this study was conducted on a small dataset, we believe our results will be useful in considering which AL algorithm to select for other studies on larger datasets, especially in recent years, when human-in-the-loop has become a hot topic.

## Supplementary Information

Below is the link to the electronic supplementary material.

Supplementary file1 (DOCX 708 KB)

## Abbreviations

NLP: Natural language processing
AL: Active learning
BERT: Bidirectional encoder representations from transformers
CT: Computed tomography
PC: Postponed case
RIC: Report importance category
UDP: Unlabeled data pool
LD: Labeled dataset
RS: Random sampling
US: Uncertainty sampling (= LC + MS + RC + ES)
LC: Least confidence
MS: Margin sampling
RC: Ratio of confidence
ES: Entropy sampling
DS: Distance-based sampling (= CD + ED)
CD: Cosine distance
ED: Euclidean distance
SS: Superiority score
AUC: Area under the receiver operating characteristic curve

## Declarations

### Human or animal participants

Statement that all human and animal studies have been approved and performed in accordance with ethical standards.
