# Supplementary material for: Comparison of active learning algorithms in classifying head computed tomography reports using bidirectional encoder representations from transformers
Source: Int J Comput Assist Radiol Surg. 2025 Jan 8;20(4):687–701. doi: 10.1007/s11548-024-03316-7 (PMC12034600; doi:10.1007/s11548-024-03316-7)
Supplement: Supplementary file 1 — Supplementary file1 (DOCX 708 KB) [file 11548_2024_3316_MOESM1_ESM.docx]

Supplemental Material


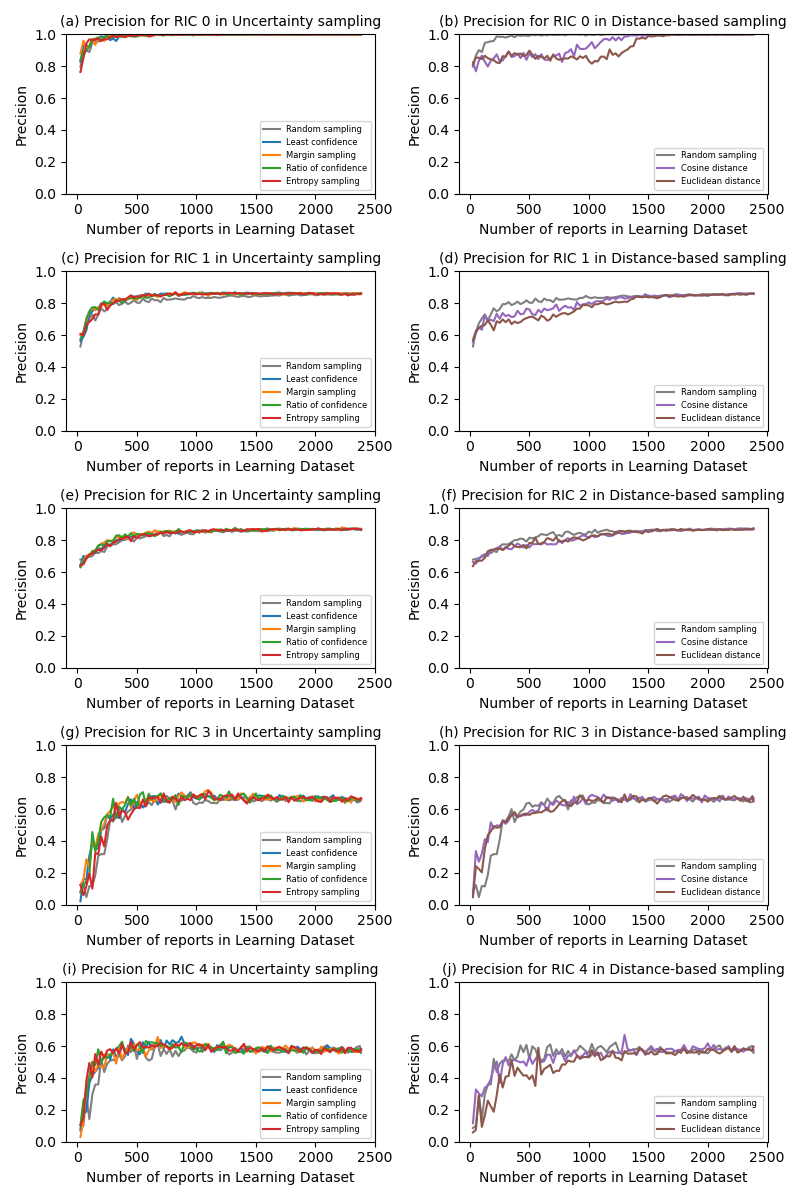


Figure S1 Transition of precision for each category


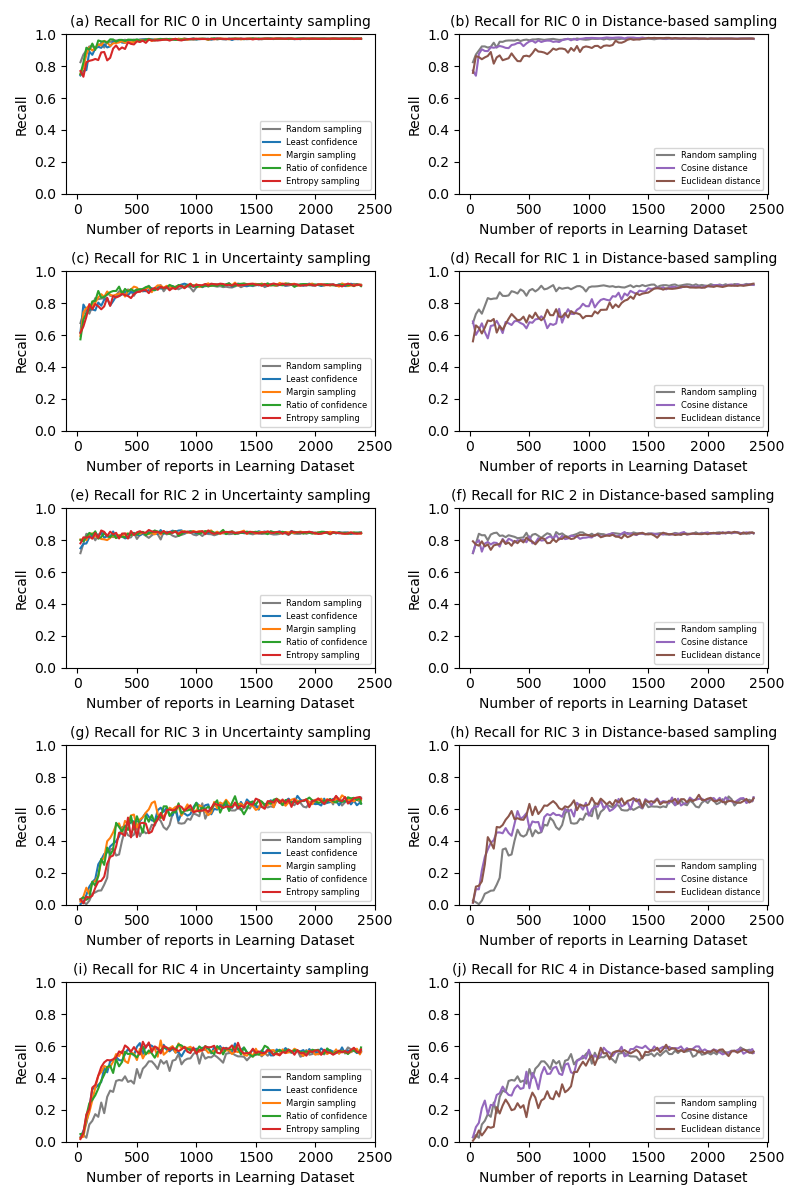


Figure S2 Transition of recall for each category


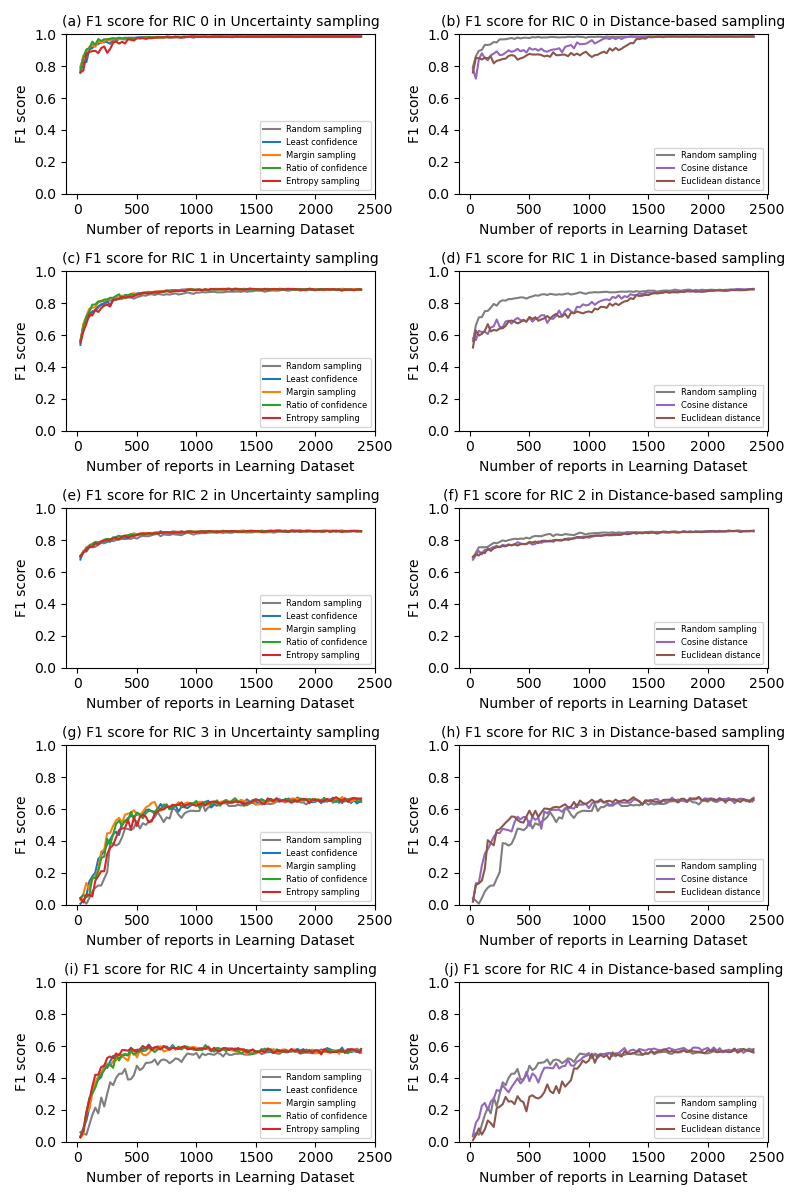


Figure S3 Transition of F1 score for each category
